# Hybridization in geese: a review

**DOI:** 10.1186/s12983-016-0153-1

**Published:** 2016-05-12

**Authors:** Jente Ottenburghs, Pim van Hooft, Sipke E. van Wieren, Ronald C. Ydenberg, Herbert H. T. Prins

**Affiliations:** Resource Ecology Group, Wageningen University, Droevendaalsesteeg 3a, 6708PB Wageningen, The Netherlands; Centre of Wildlife Ecology, Simon Fraser University, V5A 1S6 Burnaby, BC Canada

**Keywords:** Hybridization, Introgression, Behaviour, Nest parasitism, Extra-pair copulations, Fertility, Anatidae, Captivity

## Abstract

The high incidence of hybridization in waterfowl (ducks, geese and swans) makes this bird group an excellent study system to answer questions related to the evolution and maintenance of species boundaries. However, knowledge on waterfowl hybridization is biased towards ducks, with a large knowledge gap in geese. In this review, we assemble the available information on hybrid geese by focusing on three main themes: (1) incidence and frequency, (2) behavioural mechanisms leading to hybridization, and (3) hybrid fertility. Hybridization in geese is common on a species-level, but rare on a per-individual level. An overview of the different behavioural mechanisms indicates that forced extra-pair copulations and interspecific nest parasisitm can both lead to hybridization. Other sources of hybrids include hybridization in captivity and vagrant geese, which may both lead to a scarcity of conspecifics. The different mechanisms are not mutually exclusive and it is currently not possible to discriminate between the different mechanisms without quantitative data. Most hybrid geese are fertile; only in crosses between distantly related species do female hybrids become sterile. This fertility pattern, which is in line with Haldane’s Rule, may facilitate interspecific gene flow between closely related species. The knowledge on hybrid geese should be used, in combination with the information available on hybridization in ducks, to study the process of avian speciation.

## Background

Hybridization, interbreeding of species, has always intrigued ornithologists. Ernst Mayr [1] pointed out that “In birds, we have a fair amount of information, since some collectors, sensing their scarcity value, have specialized in the collecting of hybrids, and amateur observers have always been fascinated by them.” The first attempt to compile the numerous scattered references and reports of avian hybrids was undertaken by Suchetet [2]. Later on, many more checklists and compilations of avian hybrids have been published [3–10]. The incidence of hybridization varies among bird orders, with the Anseriformes (waterfowl: ducks, geese and swans) showing the highest propensity to hybridize. Over 60 % of waterfowl species has hybridized with at least one other species and this figure increases to almost 77 % when including captive hybrids [8].

The high incidence of hybridization in waterfowl makes this bird group an excellent study system to answer questions related to the origin and preservation of species. For example, how do waterfowl species remain distinct despite high levels of hybridization? Does hybridization lead to the exchange of genetic material (i.e., introgression) and if so, does this provide individuals with an adaptive advantage or disadvantage? Indeed, there are still many open questions in speciation and hybridization research that could be answered by studying hybridization in waterfowl [11, 12]. These questions, however, are not the focus of this review.

The knowledge on waterfowl hybridization is biased towards ducks, as illustrated by an extensive inventory of hybrid ducks [13], an analysis of hybrid duck fertility patterns [14] and several genetic studies documenting interspecific gene flow due to introgressive hybridization (e.g., [15–17]). The knowledge of goose hybrids is clearly lagging behind. Several studies reported goose hybrids [18–21] or provided a description of local records of hybrid geese [22–24], but no study has been dedicated to the incidence of goose hybrids or their fertility. The differences in species discrimination and social structure between ducks and geese provide the opportunity to formulate and test research questions that will broaden our understanding on the origin and preservation of waterfowl species. For instance, how does sexual selection (as measured by the degree of sexual dimorphism) relate to the frequency of hybridization? Does hybridization accelerate of slow down the speciation process? Which behavioural and morphological characateristics determine conspecific or heterospecific mate choice? Is there strong selection against hybrids?

In this review, we address the knowledge gaps on hybrid geese by focusing on three main themes: (1) incidence and frequency, (2) behavioural mechanisms leading to hybridization, and (3) hybrid fertility.

### Goose taxonomy

Table 1 gives an overview of the current taxonomic classification of the True Geese. We follow the International Ornithologists’ Union (IOU) for species names [25], with one exception. Even though IOU currently recognizes two species of Bean Goose (Taiga Bean Goose *A. fabalis* and Tundra Bean Goose *A. serrirostris*), most reports on hybridization date from before this split into two species and hence, it is not possible to analyse these Bean Goose species separately.

**Table 1:** Current taxonomy for the True Geese (tribe Anserini)

English Name	Scientific Name	Subspecies
Genus ANSER		
Swan Goose	*Anser cygnoides*	
Taiga Bean Goose	*Anser fabalis*	*A. f. fabalis* *A. f. johanseni* *A. f. middendorffii*
Tundra Bean Goose	*Anser serrirostris*	*A. serrirostris rossicus* *A. serrirostris serrirostris*
Pink-footed Goose	*Anser brachyrhynchus*	
Greater White-fronted Goose	*Anser albifrons*	*A. a. albifrons* (Eurasian) *A. a. flavirostris* (Greenland) *A. a. gambeli* (Western) *A. a. frontalis* (Western) *A. a. elgasi* (Tule)
Lesser White-fronted Goose	*Anser erythropus*	
Greylag Goose	*Anser anser*	*A. a. anser* (European) *A. a. rubrirostris* (Siberian)
Bar-headed Goose	*Anser indicus*	
Emperor Goose	*Anser canagicus*	
Snow Goose	*Anser caerulescens*	*A. c. caerulescens* *A. c. atlantica*
Ross’ Goose	*Anser rossii*	
Genus BRANTA		
Brent Goose	*Branta bernicla*	*B. b. bernicla* (Dark-bellied) *B. b. hrota* (Pale-bellied or Atlantic) *B. b. nigricans* (Black) *B. b. orientalis*
Barnacle Goose	*Branta leucopsis*	
Cackling Goose	*Branta hutchinsii*	*B. h. leucopareia* (Aleutian) *B. h. hutchinsii* (Richardson’s) *B. h. minima* (Minima) *B. h. taverneri* (Taverner’s)
Canada Goose	*Branta canadensis*	*B. c. moffitti* *B. c. maxima* *B. c. occidentalis* *B. c. fulva* *B. c. canadensis* *B. c. interior* *B. c. parvipes*
Hawaiian Goose	*Branta sandvicensis*	
Red-breasted Goose	*Branta ruficollis*	

### Incidence and frequency of goose hybrids

**Fig. 1:**
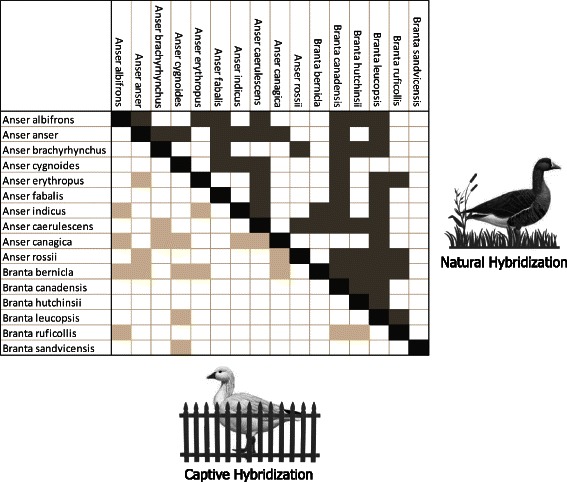
Overview of incidence of hybridization in geese. Hybridization in nature is depicted above the diagonal, whereas hybridization in captivity below the diagonal. Species that hybridized both in nature and in captivity are included only in the former category

There is an important distinction between incidence and frequency of hybridization. Incidence is binary: a certain hybrid combination has been observed or not. Figure 1 gives an overview of 74 observed hybrid geese in nature and captivity, based on records retrieved from the Serge Dumont Hybrid Database [26]. The frequency of hybridization refers to the number of hybrid individuals in the wild. Because actual numbers of hybrids are mostly not included in bird counts and some crosses are very hard to identify [27], it is nearly impossible to get an accurate estimation of the number of hybrids for certain combinations of species. However, two surveys in Great Britain monitored the frequency of hybrid geese in 1991 and 2000 when occurrence of the most common hybrid (Canada Goose x Greylag Goose) was quantified. These hybrids represent less than one per cent of the British population of Canada Geese and Greylag Geese (0.33 % in 1991 and 0.11 % in 2000) [28, 29], falling in line with previous estimates from other bird groups [13, 30–32].

Several European studies have compiled the occurrence of hybrid geese based on data from a variety of sources, such as regional and local bird magazines or personal observations (Table 2). In all studies, hybrids between Canada Goose and Greylag Goose were most numerous, while other hybrid geese were limited to a handful of individuals [23, 24, 29]. It seems that hybridization in geese is common on a species-level (Fig. 1), but rare on a per-individual level (Table 2). Although hybrids are rare in populations, a few hybrids can provide a bridge for interspecific gene flow [33], which can have important evolutionary consequences, such as adaptive introgression [34].

**Table 2:** Frequency of hybrid geese recorded in three countries: Germany [67], Great Britain [29] and Sweden [23]

Hybrid	Germany	Great Britain	Sweden
Barnacle Goose x Canada Goose	6	8	33
Barnacle Goose x Lesser White-fronted Goose	1		15
Barnacle Goose x Greylag Goose			4
Barnacle Goose x Bar-headed Goose	5	1	1
Barnacle Goose x Emperor Goose		5	
Barnacle Goose x Greater White-fronted Goose	3		1
Barnacle Goose x Red-breasted Goose	1		1
Barnacle Goose x Ross’ Goose	1		
Barnacle Goose x Snow Goose		2	
Lesser x Greater White-fronted Goose			2
Greylag Goose x Canada Goose	140	88	226
Greylag Goose x Bar-headed Goose	6	6	2
Greylag Goose x Greater White-fronted Goose		12	1
Greylag Goose x Snow Goose		20	
Greylag Goose x Swan Goose	38	57	1
Canada Goose x Bar-headed Goose	12	1	1
Canada Goose x Greater White-fronted Goose			6
Canada Goose x Swan Goose	3	4	
Bar-headed Goose x Emperor Goose		1	
Swan Goose x Bar-headed Goose	12		

### Origin of goose hybrids

Several behavioural mechanisms have been called upon to explain the production of hybrid offspring in birds [35–37]. Here, we discuss four mechanisms that are relevant for the occurrence of goose hybrids, namely (1) nest parasitism, (2) extra-pair copulations, (3) rarity of conspecifics, and (4) captive birds.

#### Nest parasitism

**Fig. 2:**
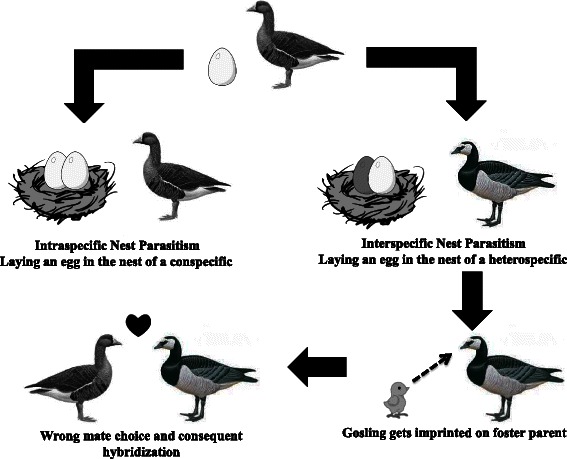
Graphical representation showing how interspecific nest parasitism can lead to hybridization

Nest parasitism and brood amalgamation occur commonly in waterfowl, both within and among species [38–40]. Intraspecific nest parasitism has been documented for several goose species (Table 3), but only three goose species are known to show interspecific nest parasitism, namely Greylag Goose, Snow Goose and Canada Goose [23, 41]. Interspecific nest parasitism could facilitate hybridization because hatching by a heterospecific foster parent might lead to sexual imprinting on the foster parent’s species and this may in turn lead to interspecific mate choice in the future (Fig. 2). The plausibility of this scenario has been assessed experimentally by means of cross-fostering experiments: Fabricius [42] placed eggs of Greylag Geese in the nest of Canada Geese. The young Greylag Geese followed their foster parents to their wintering grounds. On return, all females (16) paired with Greylag Geese, whereas 5 out of 19 males paired with Canada Geese. Furthermore, some Greylag Goose males that lost a partner remated with a female Canada Goose, showing that these males were sexually imprinted on this species.

**Table 3:** Occurrence of intra- and interspecific nestparastism and extra-pair copulations in all goose species

Species	Nest Parasitism		Extra-pair Copulations
	Intraspecific	Interspecific	
Swan Goose			
Bean Goose			
Pink-footed Goose			
Greater White-fronted Goose			[51]
Lesser White-fronted Goose			
Bar-headed Goose	[90]		
Greylag Goose	[91]	[23]	
Snow Goose	[54, 92–94]	[41]	[54, 55]
Ross’ Goose	[95]		[55]
Emperor Goose	[96]		
Hawaii Goose			
Canada Goose	[97, 98]	[41]	[53]
Barnacle Goose	[43, 46, 99]		
Brent Goose	[100, 101]		[52]
Red-breasted Goose

Some goose species adopt conspecific young [43–47]. Whether geese also adopt heterospecific goslings and if this adoption can affect sexual imprinting and future mate choice is unknown. Heterospecific adoption has been documented between several distantly related bird species, but seems to be a rare phenomenon [48].

#### Extra-pair copulations

Forced extra-pair copulations (often called “rapes”) have been reported in several species of waterfowl [49]. Trivers [50] suggested that such extrapair copulations could be functional; he noted that “a mixed strategy will be the optimal male course – to help a single female raise young, while not passing up opportunities to mate with other females whom he will not aid.” Males of several goose species engage in forced extra-pair copulations, such as Greater White-fronted Goose [51], Brent Goose [52] and Canada Goose [53]. But this behaviour has been studied most extensively in Snow Goose and Ross’ Goose [54–56]. In the Canadian Karrak Lake Colony, Dunn et al. [55] observed that among successful copulations, 33 and 38 % were extra-pair in Ross’ and Snow Geese, respectively. Despite this high precentage of extra-pair copulations, only 2–5 % of the goslings had another father than the male guarding the nest. A similar low percentage of extrapair paternity (2–4 %) was also reported for Snow Geese in northern Manitoba, Canada [54]. Based on these low ferilization percentages, forced extra-pair copulations appear to be a relatively inefficient reproductive tactic for males of these goose species. However, offspring resulting from successful extrapair copulations do provide a fitness benefit to males.

Extra-pair copulations can lead to hybridization when males copulate with females of another species. This has been observed for ducks: for instance, Seymour [57] reports three occasions of an extra-pair copulation attempt by a male Mallard (*Anas platyrhynchos*) on a female Black Duck (*A. rubripes*). However, interspecific extra-pair copulations have not been documented in geese. This can be due to the limited number of behavioural studies of geese during the period when copulations are most likely, and may also reflect differences in species discrimination and social structure between ducks and geese [49]. Male ducks often seem unable or indifferent to discriminate between females of different species (which look very similar) as many studies report male ducks displaying to heterospecific females [36, 58, 59]. The social structure of geese, with long-term pairbonds and nest guarding by males, limits the opportunties for males to seek extra-pair copulations [60]. Although interspecfic extra-pair copulations can potentially result in hybrid offspring, this behavioural mechanism seems of minor importance in the origin of hybrid geese, because of its low frequency and the low fertilization rate of such extra-pair copulations. This conclusion is in line with the study by Randler [61], who showed that “interspecific brood amalgamation has a stronger impact on natural hybridization in wildfowl than forced extra-pair copulations.”

#### Scarcity of conspecifics

Hubbs’ Principle or the Desparation Hypothesis states that the rarer species is more likely to mate with heterospecifics [62]. There are several situations in which individual birds can be confronted with a scarcity of conspecifics, such as range expansion, vagrant birds or the release/escape of captive birds in a non-native environment. With regard to geese, range expansion should include an expansion of the wintering grounds, where mate choice occurs [63]. Some birds will “make the best out of a bad job” and pair with a heterospecific mate: hybridizing with a closely related species may be a better solution than remaining unpaired [37, 64]. For instance, Indigo Buntings (*Passerina cyanea*) and Lazuli Buntings (*P. amoena*) switched to heterospecifics when no conspecific mates were available [64]. Another good example of the Desparation Hypothesis concerns two duck species on the Falkland Islands, where Speckled Teals (*Anas flavirostris*) outnumber Yellow-billed Pintails (*A. georgica*) about ten to one. This numerical imbalance leads to hybridization [65]. The Desperation Hypothesis is not restricted to natural situations, in captivity birds are often confronted with a scarcity of conspecifics and might choose to mate with the available heterospecifics.

#### Captive birds

Cockrum [3] already noted that “If hybrids resulting from birds in captivity were listed, the list would be much larger, especially among ducks and geese.” Indeed, numerous hybrids have been produced in captivity [8]. The occurrence of hybridization in captivity can be explained by the mechanisms discussed above, namely extra-pair copulations, nest parasitism and scarcity of conspecifics. When these hybrids escape, they can be mistakenly reported as wild hybrids. However, it may be possible to deduce the captive origin of hybrids when one of the parent species is not native by examining the range of occurrence. Table 2 shows that many hybrid geese probably have captive origin; for instance, some of the most common hybrids in Europe are between Greylag Goose and two introduced species, Canada Goose and Swan Goose. However, there is also the possibility that vagrant geese enter the range of other species. For example, North American Snow Geese are occasionally observed in Europe during migration [66] and hybrids between Snow Goose and several European species have been reported [10].

Randler [67] introduced the “captivity effect” to account for the high rates of *Anser* hybrids in released populations. He argued that domestication of Greylag Goose and Swan Goose has resulted in genetical impoverishment and unnatural behaviour, leading to a relatively strong tendency for hybridization. For example, in Greylag Geese, the frequency of hybrids was higher in naturalised compared to natural populations [23, 67, 68]. The effects of captivity on hybridization should thus be taken into account.

### Fertility of goose hybrids

In *The Origin of Species*, Darwin [69] discussed the fertility of hybrids between two domesticated goose species, the Greylag Goose and the Swan Goose:

“The hybrids from the common and Chinese geese (*A. cygnoides*), species which are so different that they are generally ranked in distinct genera, have often bred in this country with either pure parent, and in one single instance they have bred inter se. This was effected by Mr Eyton, who raised two hybrids from the same parents but from different hatches; and from these two birds he raised no less than eight hybrids (grandchildren of the pure geese) from one nest. In India, however, these cross-bred geese must be far more fertile; for I am assured by two eminently capable judges, namely Mr Blyth and Capt. Hutton, that whole flocks of these crossed geese are kept in various parts of the country; and as they are kept for profit, where neither pure parent-species exists, they must certainly be highly fertile.”

Later, he repeated the experiment of Mr. Eyton by crossing “a brother and sister hybrid from the same hatch” that he received from Rev. Goodacre [70]. He only managed to rear five hybrids (several eggs did not hatch or remained unfertilized), but he was still startled by “the fact that these two species of geese [are] breeding so freely together.” He attributed the fertility of these hybrids to the long history of goose domestication. We now know that, irrespective of domestication, the potential for hybridization is lost slowly on an evolutionary timescale in birds, [71] and that many bird species are capable of producing fertile hybrids [72].

The evolution of hybrid sterility and inviability (both caused by postzygotic incompatibilities) has been studied in *Drosophila* [73], frogs [74], butterflies [75] and birds [72]. These studies showed an increase of postzygotic isolation between species with divergence time. Furthermore, the evolution of postzygotic incompatibility follows Haldane’s Rule [76], which states that “when in the F_1_ offspring of two different animal races one sex is absent, rare, or sterile, that sex is the heterozygous [or heterogametic] sex”. In birds, where sex is determined by a ZZ/ZW system, females are the heterogametic sex and hybrid females are thus expected to show greater fitness reductions compared to male hybrids. This expectation has been confirmed for birds in general [72], but also for specific bird groups, including ducks [14], galliform birds [77] and pigeons and doves [78].

One of the possible mechanisms that has been invoked to explain Haldane’s rule is dominance theory, which is based on the Dobzhansky-Muller incompatibility model [79, 80]. Dominance theory states that hybrid sterility and unviability are the outcome of the interaction of two (or more) genes that have developed incompatible alleles in allopatry. If these alleles are recessive and located on the Z-chromosome, their effect will be much larger in female birds because this sex lacks another Z-chromosome that could hold a dominant version of the incompatible allele, which would nullify the negative effect of the recessive one. Moreover, it has been suggested that the Z-chromosome plays a disproportionately large role in the development of intrinsic incompatibilities [81]. Several lines of evidence support this “Large Z-effect.” First, Z-linked genes evolve faster compared to autosomal loci (“Faster Z-effect”), thereby speeding up the accumulation of incompatible alleles on this sex chromosome [82, 83]. Second, if genes involved in premating and postzygotic isolation both occur on the Z-chromosome and thus become physically linked, it is expected that this facilitates the evolution of isolation barriers by means of reinforcement [84]. This situation has been described for *Ficedula* flycatchers, where genes for female preference and low hybrid fitness are located on the Z-chromosome [85, 86].

We tested whether geese also conform to Haldane’s Rule. We obtained cytochrome b sequences from GENBANK and calculated genetic distances between taxa using the Maximum Composite Likelihood model with Gamma Distribution in MEGA6 [87]. Reports on hybrid goose fertility were collected from the Handbook of Avian Hybrids of the World [10]. We performed a logistic regression in SPSS (version 19.0) with hybrid fertility as dependent variable (0 = both sexes fertile, 1 = only males fertile) and genetic distance as independent variable. To our knowledge, there are no reports of goose hybrids where only the females are fertile.

**Fig. 3:**
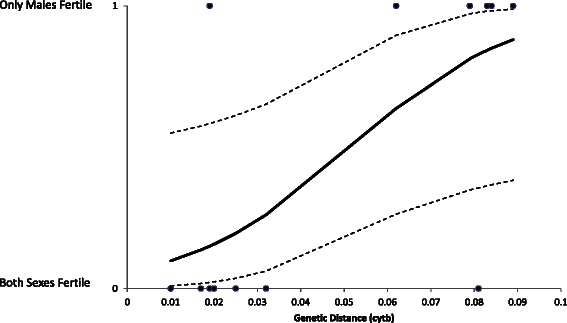
Fertility of goose hybrids at different genetic distances (based on cytochrome b sequences). At high genetic distances only male hybrids are fertile, a pattern in accordance with Haldane’s Rule

At high genetic distances, for most species only male hybrids are fertile (Fig. 3, *ß* = 53.425, SE = 25.485, *z-value* = 2.096, *p* = 0.0361), a pattern that is consistent with Haldane’s Rule. Two species pairs deviate from the expected pattern: only male hybrids between the congeneric Greater White-fronted Goose and Swan Goose are fertile and both sexes are fertile when crossing the more distantly related Canada Goose and Greater White-fronted Goose. However, a more detailed analysis is necessary to fully understand the evolution of postzygotic incompatibilities in geese. For example, Lijtmaer et al. [78] studied postzygotic isolation in pigeons and doves based on records of old interspecific breeding experiments [88], that included data on the number of unhatched eggs and the sex ratio of clutches. Such analyses provide insights, not only into the fertility of hybrids, but also into the fertility and viability of backcrosses. For instance, Arrieta et al. [77] showed that hybrid inviability was higher in F_2_ compared to F_1_ hybrids in galliform birds, indicating that interspecific gene flow may be hampered due to inviable F_2_ hybrids. For geese, the fertility of male birds at high genetic distances suggests the possibility of interspecific gene flow between distantly related species (e.g., Greylag Goose and Canada Goose), but if consequent backcrosses are sterile or inviable, then the possibility of interspecific gene flow is greatly reduced. On the other hand, the fertility of hybrids at low genetic distances (e.g., Greater White-fronted Goose and Greylag Goose) provides the opportunity of interspecific gene flow between closely related species. For example, Leafloor et al. [89] reported gene flow between Canada Goose and Cackling Goose across an arctic hybrid zone.

## Conclusions

Hybridization in geese is common on a species-level, but rare on a per-individual level. The origin of the occasional hybrids is difficult to determine. An overview of the different mechanisms shows that, in theory, interspecific nest parasisitm or forced extra-pair copulations could lead to hybridization. Other sources of hybrids include a scarcity of conspecifics, hybridization in captivity and vagrant geese. The different mechanisms are not mutually exclusive, for instance, certain hybrids might be the result of extra-pair copulations in captivity. Currently, it is not possible to discriminate between the different mechanisms without quantitative data. To unravel the relative importance of these mechanisms, field data should be collected and experiments could be conducted in captivity. For example, the frequency of interspecific nest parasitism and extra-pair copulations may be documented in mixed breeding colonies. The occurrence of possible hybrids (which can be identified by means of genetic tests) in such colonies can then be related to the frequency of these behaviours. In captivity, experiments can be set up to observe how different goose species react to a scarcity of conspecifics and the availability of diverse heterospecifics. Most goose hybrids are fertile; only at high genetic distances do female hybrids become sterile. This fertility pattern provides the opportunity for interspecific gene flow between closely related species.

The overview of hybridization in geese presented here can be used, in combination with the knowledge available on duck hybrids, to study the process of avian speciation. Moreover, the differences in species discrimination and social structure between ducks and geese provide the opportunity to formulate and test research questions that will broaden our understanding on the origin and preservation of species.
